# An NMF-L_2,1_-Norm Constraint Method for Characteristic Gene Selection

**DOI:** 10.1371/journal.pone.0158494

**Published:** 2016-07-18

**Authors:** Dong Wang, Jin-Xing Liu, Ying-Lian Gao, Jiguo Yu, Chun-Hou Zheng, Yong Xu

**Affiliations:** 1 School of Information Science and Engineering, Qufu Normal University, Rizhao, 276826, China; 2 Library of Qufu Normal University, Qufu Normal University, Rizhao, 276826, China; 3 Bio-Computing Research Center, Shenzhen Graduate School, Harbin Institute of Technology, Shenzhen, 518055, China; Brown University, UNITED STATES

## Abstract

Recent research has demonstrated that characteristic gene selection based on gene expression data remains faced with considerable challenges. This is primarily because gene expression data are typically high dimensional, negative, non-sparse and noisy. However, existing methods for data analysis are able to cope with only some of these challenges. In this paper, we address all of these challenges with a unified method: nonnegative matrix factorization via the *L*_2,1_-norm (NMF-*L*_2,1_). While *L*_2,1_-norm minimization is applied to both the error function and the regularization term, our method is robust to outliers and noise in the data and generates sparse results. The application of our method to plant and tumor gene expression data demonstrates that NMF-*L*_2,1_ can extract more characteristic genes than other existing state-of-the-art methods.

## 1 Introduction

The development of microarray technologies makes the study of complex biological gene expression networks possible. Microarray datasets typically contain expression data for the thousands of genes profiled on each chip, and the number of replicates is much smaller than the number of genes, making the selection of genes difficult[1]. In addition, the inclusion of irrelevant or noisy variables may decrease the accuracy of selection[2]. The problem of how to select genes associated with the target terms has become a challenge for scientists[3]. For example, plants are able to cope with environmental challenges such as cold, heat, and salt, which are referred to as abiotic stresses; there must therefore exist specific interacting genes that respond to each abiotic stress. Another typical example is that of cancer, an important cause of human morbidity; the identification of genes that are frequently mutated in cancers and play an essential role in cancer development is critical. Many methods have been proposed for processing gene expression data collected by DNA microarray profiling[4–9]. For example, Liu et al.[10]used a method based on penalized matrix decomposition (PMD) to extract characteristic plant genes, and Zheng et al.[11] applied nonnegative matrix factorization (NMF) to tumor gene selection. Principal component analysis (PCA) and singular value decomposition (SVD) have also been used to analyze gene expression data[12]. Liu et al.[13] proposed a CIPMD algorithm (A Class-Information-Based Penalized Matrix Decomposition) for identifying plants core genes responding to abiotic stresses. This method is PMD method with label information. Liu et al.[14] proposed a PRFE algorithm (A P-Norm Robust Feature Extraction) for identifying differentially expressed genes. Although those methods are all feature selection methods and in widespread use, they present some disadvantages:

1. Although the elements of the initial data matrix are entirely nonnegative, the traditional low-rank algorithm [15]cannot guarantee nonnegative values in the project matrix, thereby complicating their biological interpretation.
2. The high dimensionality of data poses challenges, such as the so-called curse of dimensionality[16,17].
3. Faced with millions of individual data points, it is difficult to interpret gene expression data without sparse constraints.
4. Gene expression data often contains numerous outliers and abundant noise, which traditional methods do not effectively address.

NMF has been widely used in various fields because it can generate low-rank and nonnegative results. The ability to generate a low-rank nonnegative matrix to approximate a given nonnegative data matrix is a significant advantage[18], but the lack of sparsity in data processed via NMF makes this method less than ideal for characteristic gene selection. In high throughput datasets, gene expression data are high dimensional and always contain some redundant information(i.e., not all features are relevant). To address these problems, we sought to incorporate sparsity, or the reduction of certain vector elements to zero. The regular inclusion of sparsity has played a significant role in dimensionality reduction and feature selection[19]. For example, Journée et al.[20] proposed a sparse principal component analysis (SPCA) method using the generalized power method, and Wittenet al.[21]proposed a penalized matrix decomposition (PMD) method, which has been proven useful in microarray analysis by imposing penalization on factor matrices. Nonnegative matrix factorization with sparse constraints (NMFSC), which was first introduced by Patrik O. Hoyer in 2004 [22], accurately controls sparsity. NMFSC has been applied to the problems of imaging and gene selection, among others. However, it does not guarantee that entire rows of a matrix are sparse, which can lead to difficulties during feature selection. To address these issues, the *L*_2,1_ version of NMF favors the inclusion of a small number of non-zero rows in the factor matrix, which are proposed to generate sparse results for rows.

However, these methods for generating sparsity apply the least square error function, which does not reliably address noise and outliers [23]. When faced with these complications, the error for both features and samples will be squared[24], increasing the effect of large noises or outliers [25]. As a result, the *L*_2,1_ version of the error function has been proposed to address noisy data [26].

In light of these problems, we propose a novel method called Nonnegative Matrix Factorization with *L*_2,1_-norm (NMF-*L*_2,1_), which imposes an *L*_2,1_-norm constraint on both the error function and a regularization term to solve the aforementioned problems simultaneously. A sparse regularization term avoids the potential problem of over-fitting and selects a sparse subset of features. Rather than use an *L*_2_-norm-based error function, the *L*_2,1_-norm-based error function diminishes the impact of the outliers and noise in a dataset[25,27].

The main contributions of this paper are the following:

First, *L*_2,1_-norm is employed for regularization in our method to generate sparse results, making the results easier to interpret.
Second, nonnegative matrix factorization is utilized to generate low-rank results with nonnegative values.
Third, the *L*_2,1_-norm-based error function is used to diminish the outliers and noise inherent in gene expression data.

This paper is organized as follows. The methodology section introduces the NMF-*L*_2,1_ method and provides an efficient algorithm for estimation. The results and discussion section compares our method with other three methods: PMD, NMFSC and SPCA. Our conclusions are presented in the third section.

## 2 Methodology

### 2.1 Mathematical Definition of L_2,1_-norm

This subsection briefly introduces the *L*_2,1_-norm proposed in [28]. It is defined as

$$\|M{\|}_{2,1}=\sum_{\text{i}=1}^{\text{n}}\sqrt{\sum_{j=1}^{\text{s}}m_{ij}^{2}}=\sum_{i=1}^{n}\|m^{i}{\|}_{2},$$ (1)

where **m**^*i*^ is the i-th row of **M**, *m*_*ij*_ is the (*i*, *j*)-th entry in **M**, **M** is an *n* × *s* matrix. An explanation of *L*_2,1_-norm is as follows. First, we compute the *L*_2_-norm of rows **m**^*i*^, then compute the *L*_1_-norm of vector *b*(**M**) = (‖**m**^1^‖_2_,‖**m**^2^‖_2_,…,‖**m**^*s*^‖_2_). The amplitude of the components of vector b(**M**) dictate how important each dimension is *L*_2,1_-norm favors a small number of non-zero rows in **M**,ensuring that an appropriate dimensional reduction is achieved[29].

### 2.2 Extracting Characteristic Genes by NMF-L2,1

In this paper, the matrix **X** denotes the initial gene expression dataset, whose size is *n* × *c*. Each column of **X** represents the transcriptional response of the *n* genes in one sample. Each row of **X** represents the expression level of a gene across all samples. Thus, the **X** can be approximated as:

X≈AY, (2)

where **A** is an *n* × *d* matrix, **Y** is a *d* × *c* matrix, and *d* < min(n, c).

The matrices **Y** and **A** are called the coefficient and basis matrices, respectively. Given suitable parameters for NMF-*L*_2,1_, the sparse matrix **A** can be obtained. Characteristic genes can then be extracted according to the non-zero entries in **A** [30].

### 2.3 NMF based on L2,1-norm (NMF-L2,1)

Let **X** = (**x**_1_, **x**_2_,…,**x**_*c*_) ∊ *R*^*n*×*c*^, **Y** = (**y**_1_, **y**_2_,…,**y**_*c*_)^*T*^ ∊ *R*^*d*×*c*^. The error function of standard NMF [31] is

$$\|X-AY{\|}_{F}^{2}=\sum_{i=1}^{c}\|x_{i}-Ay_{i}{\|}^{2},\begin{array}{cc} & st. \end{array}\;Y>0,A>0.$$ (3)

Here, the error for each data point is calculated as a squared residual error in terms of ‖**x**_*i*_−**Ay**_*i*_‖^2^. As a result, a few outliers with large errors can easily dominate the objection function due to the squared errors. Thus, it is reasonable to propose an NMF-*L*_2,1_ formulation to reduce the influence of outliers and errors.

The error function of the NMF-*L*_2,1_ formulation is:

$$\|X-AY{\|}_{2,1}=\sum_{i=1}^{c}\sqrt{\sum_{j=1}^{n}{\left(X-AY\right)}_{ij}^{2}}=\sum_{i=1}^{c}\|x_{i}-Ay_{i}\|.$$ (4)

In this formulation, the error for each data point is ‖**x**_*i*_−**Ay**_*i*_‖, which is not squared; thus, the impact of large errors caused by outliers does not fully dominate the objective function[32].

The NMF-*L*_2,1_ optimization problem is formulated as

$$\underset{Y}{\text{min}}\|X-AY{\|}_{2,1}+\lambda \|Y{\|}_{2,1},\begin{array}{cc} & st. \end{array}\;Y>0,A>0.$$ (5)

The problem in Eq (4) is equivalent to

$$\underset{Y,E}{\text{min}}\|E{\|}_{2,1}+\|Y{\|}_{2,1},\begin{array}{cc} & st. \end{array}\;AY+\lambda E=X,Y>0,A>0.$$ (6)

Thus, the above problem can be rewritten as

$$\begin{array}{cc} \underset{Y,E}{\text{min}}\|\left[\begin{array}{l} Y\\ E \end{array}\right]{\|}_{2,1}, & \begin{array}{cc} & st.\;\left[\begin{array}{cc} A & \lambda I \end{array}\right]\left[\begin{array}{l} Y\\ E \end{array}\right]=X,Y>0 \end{array} \end{array},A>0,$$ (7)

where **I** ∊ *R*^n×*n*^ is an identity matrix, *n* = *c*. Let **B** = [**A** *λ***I**] ∊ *R*^n×*b*^ and $U={\left[\begin{array}{cc} Y^{T} & E^{T} \end{array}\right]}^{T}\in R^{b\times c}$, where *b* = *d* + *n*.

Then, the problem can be reformulate das

$$\underset{U>0}{\text{min}}\|U{\|}_{2,1},\begin{array}{cc} & st.\;BU=X \end{array}.$$ (8)

How can this optimization problem handle high dimensional, nonnegative, noisy and sparse data simultaneously? The reasons are as follows:

1. The *L*_2,1_-norm error function term is designed to diminish the impact of noise or outliers contained in the original data. As a result, we can expect to obtain cleaner data for subsequent analyses.
2. This clean data may be not sparse–some features may be irrelevant to the learning procedure—so the *L*_2,1_-norm regularization term[33]can be used to generate a sparse solution.
3. In the next section, we will demonstrate that the above conditions produce more effective models, especially for datasets that are sparse, nonnegative, high dimensional and noisy.

### 2.4 An Efficient Algorithm for NMF-L2,1

To solve the constrained optimization problem in Eq (7), Nie et al.[34]have provided an efficient algorithm. Here, we briefly introduce the efficient algorithm.

By introducing Lagrangian multiplier **Λ**, we first give the Lagrangian function as follows:

$$L(U)=\sum_{i=1}^{\text{b}}\|u^{i}{\|}_{2}-\text{Tr}(\Lambda^{T}(BU-X)),$$ (9)

where Tr() is the trace function of a matrix. Here we introduce the augmented cost-function

$$J(U,q)=\text{Tr}(U^{T}QU)-\text{Tr}(\Lambda^{T}(BU-X)),$$ (10)

where **q** ∊ *R*^*b*^ is an auxiliary vector and **Q** = *diag*(**q**) is a diagonal matrix with the diagonal element

$$Q=\text{diag}(q)=diag\left(\frac{1}{2\|u_{}^{i}\text{+}\varepsilon {\|}_{2}}\right),$$ (11)

in which *ε* is a positive number and infinitely close to, but not equal to, zero.

Taking the derivative of *J*(**U**, **q**) with respect to **U** to zero, we obtain:

$$\frac{\partial J(U,q)}{\partial U}=2QU-B^{T}\Lambda =0.$$ (12)

By multiplying the two sides of Eq (11) by **BQ**^−1^ and using the constraint **BU** = **X**, we obtain

$$\begin{array}{l} 2BU-BQ^{-1}B^{T}\Lambda =0\\ \to 2X-BQ^{-1}B^{T}\Lambda =0\\ \to \Lambda =2{\left(BQ^{-1}B^{T}\right)}^{-1}X. \end{array}$$ (13)

Then, we obtain:

$$U=Q^{-1}B^{T}{\left(BQ^{-1}B^{T}\right)}^{-1}X.$$ (14)

More details of the algorithm can be found in[34]. Here, we summarize our method in Box 1. In each iteration, **U** is calculated with the current **Q**. Then, **Q** is updated based on the current **U**. The iteration procedure is repeated until the algorithm converges.

**Box 1. NMF-L_2,1_ method.**

Input: **X** ∊ *R*^*n*×*c*^ and parameter λ.

Output: **Y** ∊ *R*^*d*×*c*^,**A** ∊ *R*^*n*×*d*^.

1: Initialize **Q**_t_ ∈ *R*^*b*×*b*^ as an identity matrix and **A** ∊ *R*^n×*d*^ as a nonnegative matrix,

 set t = 0.

2: repeat

 Compute $U_{t+1}=Q_{t}^{-1}B^{T}{\left(BQ_{t}^{-1}B^{T}\right)}^{-1}X$.

 Setting **U** > **0**.

 Compute diagonal matrix **Q**_t+1_ according to Eq (11).

 t = t+1.

 **A** and **Y** are obtained from **U** according to Eq (7).

Until convergence.

In this paper, the characteristic genes are extracted by the coefficient matrix **A**. We summarize the NMF-*L*_2,1_ method to extract core genes as follows:

1. Create the data matrix **X** based on gene expression data.
2. Obtain the basis matrix **A** by using the NMF-*L*_2,1_ method.
3. Extract characteristic genes from non-zero entries in matrix **A**.
4. Exploit the Gene Ontology (GO) tool to investigate the extracted genes.

## 3 Results and Discussion

In this section, several experiments are carried out. In the first subsection, the NMF-*L*_2,1_ method is compared with the following methods for a gene expression dataset obtained from plants responding to abiotic stresses: (a) the PMD method (proposed by Witten et al. [15]); (b) the SPCA method (proposed by Journée et al. [35]); and (c) the NMFSC method (proposed by Patrik O. Hoyer[36]);(d) the CIPMD method (proposed by Liu et al.[13]); (e) PRFE method (proposed by Liu et al. [14]). In the second subsection, the six methods are compared for Medulloblastoma and leukemia tumor datasets.

### 3.1 Results for Plant Gene Expression Data

Plants are continually challenged by environmental parameters such as drought, salt, cold, osmotic pressure, and UV-B light [37]. Among plant genes, there must exist a specific set of interacting genes that respond to each abiotic stress. Thus, it is important but challenging to extract genes responding to each abiotic stress from plant gene expression data.

#### 3.1.1 Data source

The gene expression datasets used in our experiment were downloaded from the *NASC* Arrays [http://affy.arabidopsis.info/link_to_iplant.shtml]. Each sample profiles 22810 genes. The plant gene expression datasets are shown in supplementary file (S1 Table). Table 1 lists the reference numbers and sample numbers for each stressor.

**Table 1:** Reference and Sample Numbers for Stress Types.

Stress type	Drought	Salt	UV-B	Cold	Heat	Osmotic	Control
Reference Number	141	140	144	138	146	139	137
Sample Number	6	7	6	7	8	6	9

#### 3.1.2 The selection of parameter λ

In order to obtain the most effective results, we used gene expression data to train the parameter λ. For each sample, the parameter varied from 0–1 with a step of 0.1, and GO Terms were used to select the most appropriate parameter. The results are provided in Table 2.

**Table 2:** The Selection of Parameter λ.

Stress type	Drought	Salt	UV-B	Cold	Heat	Osmotic
Shoot	0.3	0.3	0.2	0.5	0.3	0.3
Root	0.3	0.3	0.3	0.3	0.3	0.3

#### 3.1.3 gene ontology (GO) analysis

In this paper, GO Term is used to evaluate the genes that responded to plant abiotic stressors[38]. GO Term Finder analysis provided information to aid with the biological interpretation of high-throughput experiments. GO Term Finder is available publicly at [http://go.princeton.edu/cgi-bin/GOTermFinderS] [39]; it aims to describe genes in the query/input set and to find the genes that may have something in common.

For the sake of simplicity, 500 genes were selected from the gene expression data by the NMFSC, PMD, SPCA, CIPMD, PRFE and NMF-*L*_21_ methods. The threshold parameters used were: maximum P-value = 0.01, and minimum number of genes = 2.

#### 3.1.4 Response to stimulus

Table 3 summarizes the results of the response to a stimulus whose background frequency in the TAIR (A.thaliana (common wallcress))set was 6617/30320 (21.8%). The results are presented according to P-value and sample frequency. The P-value was calculated using a hyper-geometric distribution (details can be seen in[40]). The sample frequency denotes the number of the characteristic genes selected. For example, 330/500 denotes that 330 genes corresponding to GO terms out of 500 genes were selected by the method.

**Table 3:** Response to Stimulus (GO: 0050896).

Stress type	NMF-L21		NMFSC		PMD		SPCA		CIPMD		PRFE	
	P-value	Sample frequency	P-value	Sample frequency	P-value	Sample frequency	P-value	Sample frequency	P-value	Sample frequency	P-value	Sample frequency
Drought s	**2.77E-122**	**353/500 70.60%**	5.28E-109	342/500 68.40%	1.09E-55	276/500 55.20%	1.19E-55	273/500 54.60%	9.08E-106	338/50067.7%	3.39E-93	314/50062.8%
Drought r	2.69E-69	293/500 58.60%	2.39E-88	318/500 63.60%	3.67E-65	287/500 57.40%	5.27E-65	289/500 57.70%	**5.54E-101**	**333\50066.6%**	3.49E-41	240/50048%
Salt s	7.71E-53	271/500 54.20%	2.01E-52	268/500 53.70%	3.78E-48	262/500 52.40%	3.31E-21	262/500 52.40%	**2.00E-81**	**309/50061.9%**	1.72E-39	236/50047.5%
Salt r	**5.64E-96**	**326/500 65.20%**	3.69E-94	325/500 65.00%	1.25E-80	314/500 62.80%	1.42E-34	237/500 47.40%	1.31E-70	295/50059%	2.95E-55	163/50052.6%
UV-B s	**6.26E-152**	**382/500 76.40%**	1.62E-126	360/500 72.00%	4.99E-128	362/500 72.40%	2.59E-33	332/500 66.40%	1.81E-103	335/50067.3%	7.72E-89	308/50061.8%
UV-B r	5.85E-42	247/500 49.40%	3.81E-64	286/500 57.20%	1.21E-36	242/500 48.20%	9.56E-22	210/500 42.50%	**6.85E-95**	**326/50065.2%**	3.16E-34	227/50045.5%
Cold s	1.61E-85	312/500 62.40%	3.81E-77	304/500 60.80%	1.58E-66	294/500 58.80%	4.31E-62	283/500 56.60%	**7.52E-98**	**329/50065.9%**	1.10E-47	250/50050.3%
Cold r	2.56E-81	309/500 61.80%	7.59E-79	306/500 61.20%	8.44E-72	291/500 58.20%	3.08E-61	281/500 56.20%	**6.92E-105**	**337/50067.5%**	7.84E-46	248/50049.6%
Heat s	3.43E-25	218/500 43.60%	4.28E-19	204/500 40.80%	2.56E-23	220/500 44.00%	2.56E-23	300/500 46.40%	**2.39E-98**	**330/50066.0%**	9.16E-31	220/50044.2%
Heat r	3.96E-23	214/500 42.80%	3.22E-16	197/500 39.40%	1.18E-19	205/500 41.0%	1.69E-17	200/500 40.00%	**7.27E-105**	**337/50067.5%**	2.10E-14	182/50036.6%
Osmotic s	5.96E-73	298/500 59.60%	1.02E-82	311/500 62.20%	3.07E-75	294/500 58.80%	4.20E-49	263/500 52.60%	**6.15E-92**	**322/50064.5%**	2.17E-51	257/50051.4%
Osmotic r	5.84E-55	273/500 54.40%	1.65E-47	260/500 52.10%	2.67E-21	221/500 44.20%	1.12E-34	237/500 47.50%	**2.54E-76**	**302/50060.2%**	9.00E-25	208/50041.6%

‘s’ denotes shoot samples; ‘r’ denotes root samples.

As listed in Table 3, the six methods were compared by sample frequency and P-value. NMFSC, NMFL21, PRFE, SPCA and PMD are unsupervised methods, so we first compare the five algorithms. In the 12 terms, the results show that our method could extract more characteristic genes than the other methods for eight of twelve samples. For example, for shoot samples exposed to UV-B stress, the sample frequency was 76.4% by our method, 72% by NMFSC, 72.4% by PMD, 66.4% by SPCA and 61.8% by PRFE. This shows that our method is markedly improved over PRFE, PMD and SPCA. When compared with the supervised method CIPMD, except the salt and UV-B stress, our methods performs worth than CIPMD. Generally speaking, since supervised methods take the class labels into consideration, they usually have better performance than unsupervised methods.

#### 3.1.5 Response to the abiotic stimulus

Table 4 summarizes the results of the six methods for datasets describing the response to abiotic stimulus whose background frequency in the TAIR (A.thaliana (common wallcress)) set is 1539/29556 (5.2%). The numbers of characteristic genes and the P-values of genes responding to an abiotic stimulus (GO:0009628) in root and shoot samples are listed in Table 4.

**Table 4:** Response to an Abiotic Stimulus (GO:0009628).

Stress type	NMF-L_21_		NMFSC		PMD		SPCA		CIPMD		PRFE	
	P-value	Sample frequency	P-value	Sample frequency	P-value	Sample frequency	P-value	Sample frequency	P-value	Sample frequency	P-value	Sample frequency
Drought s	6.32E-59	**182/50036.4%**	4.89E-36	149/500 29.80%	3.91E-34	107/500 21.40%	7.50E-21	87/500 17.00%	2.28E-50	170/50034%	4.85E-28	136/50027.2%
Drought r	1.82E-22	126/500 25.20%	3.65E-23	127/500 25.40%	1.78E-10	68/500 13.60%	4.14E-08	63/500 12.60%	1.21E-55	177/50035.5%	8.05E-60	**183/50036.6%**
Salt s	2.71E-50	**170/500 34.00%**	9.95E-43	159/500 31.80%	9.93E-39	113/500 22.60%	9.83E-33	105/500 21.00%	5.65E-44	161/50032.2%	1.00E-39	114/50022.8%
Salt r	7.32E-36	149/500 29.80%	8.58E-47	165/500 33.00%	1.36E-15	78/500 15.60%	6.18E-12	71/500 14.00%	5.52E-57	**295/50059%**	7.90E-39	153/50030.8%
UV-B s	9.11E-46	164/500 32.80%	4.95E-49	168/500 33.80%	1.76E-13	74/500 14.80%	7.84E-23	90/500 18.00%	7.04E-55	**176/50035.3%**	1.53E-27	135/50027.1%
UV-B r	1.17E-14	110/500 22.00%	2.75E-22	100/500 20.00%	5.30E-10	67/500 13.40%	8.00 E-4	52/500 10.00%	5.20E-61	**184/50036.9%**	4.30E-51	171/50034.3%
Cold s	5.78E-64	**188/50037.60%**	3.85E-61	183/500 36.60%	5.82E-35	106/500 21.60%	1.17E-19	85/500 17.00%	8.99E-52	172/50034.4%	5.02E-56	178/50035.6%
Cold r	1.05E-53	175/50035.00%	1.59E-52	173/500 34.60%	2.74E-23	91/500 18.20%	4.10E-19	84/500 16.80%	6.31E-58	180/50036.1%	4.24E-61	**184/50037.0%**
Heat s	7.16E-22	125/50025.00%	3.43E-76	118/500 23.60%	1.44E-24	93/500 18.60%	4.64E-22	89/500 17.80%	1.13E-52	**173/50034.6%**	1.37E-35	148/50029.8%
Heat r	9.27E-34	145/50029.00%	6.04E-32	142/500 28.40%	1.41E-15	78/500 15.60%	1.35E-08	64/500 12.80%	1.64E-52	**173/50034.6%**	1.18E-49	169/50033.9%
Osmotic s	6.35E-56	178/500 35.60%	8.22E-61	**184/500 36.80%**	6.55E-38	112/500 22.40%	2.02E-18	83/500 16.60%	4.88E-47	165/50033.1%	4.76E-28	136/50027.2%
Osmotic r	1.32E-49	169/50033.80%	2.01E-39	154/500 30.80%	1.40E-14	76/500 15.20%	2.87E-17	81/500 16.20%	1.28E-47	166/50033.3%	1.67E-54	**176/50035.2%**

‘s’ denotes shoot samples; ‘r’ denotes root samples.

As described above in the ‘Response to stimulus’ section, we first compare the five algorithms NMFSC, NMFL21, PRFE, SPCA and PMD. From the results we can see that our method could extract more characteristic genes than the PMD and SPCA methods for all datasets. For the four sample datasets (drought, salt, UV-B and osmotic), our method performed worse than NMFSC, but superior to PMD and SPCA. When compared with the supervised method CIPMD, except the salt, heat and UV-B stress, CIPMD performs better than other methods.

#### 3.1.6 Characteristic terms

In Table 5, we list the characteristic terms. Our method outperformed SPCA and PMD for all 12 items and outperformed NMFSC for seven items. Only in one item (shoot sample in UV-B) the PRFE outperforms than our method. However, for one of the twelve items (cold in root) our method produced the same result as NMFSC. From these results, it can be concluded that our method is more effective than other unsupervised methods. The response to water deprivation (GO:0009414) for shoot samples is also analyzed in Table 5. The background frequency of the response to water deprivation (GO: 0009414) is 1.4%. It is obvious that NMF-*L*_2,1_ is able to extract more characteristic genes than the other methods, and the sample frequency in response to water deprivation by our method is 18.1%, while it is 13.2% for NMFSC, 11.9% for PMD, 16.8% for CIPMD, 11.2% for PRFE and 8.2% for SPCA, indicating that our method performs 6.2% better than PMD, 7% better than PRFE and almost 10% better than SPCA.

**Table 5:** Characteristic Terms Selected from GO by Algorithms.

Stress type	GO Terms	Background frequency	Sample frequency and ratio					
			NMF-L21	NMFSC	PMD	SPCA	CIPMD	PRFE
Drought s	GO:0009414 response to water deprivation	207/298870.70%	**91/50018.20%**	66/500 13.20%	47/500 9.40%	23/500 4.60%	84/50016.8%	56/50011.2%
Drought r	GO:0009415 response to water deprivation	207/298870.70%	58/500 11.60%	62/500 12.40%	26/500 5.20%	24/500 4.80%	**69/50013.8%**	30/5000.6%
Salt s	GO:0009651 response to salt stress	395/29887 1.30%	**80/500 16.00%**	80/500 16.00%	41/500 8.20%	28/500 5.60%	64/50012.8%	42/5008.4%
Salt r	GO:0009651 response to salt stress	395/298871.30%	74/500 14.80%	**76/500 15.20%**	33/500 6.60%	22/500 4.40%	57/50011.4%	37/5007.4%
UV-B s	GO:0009416 response to light stimulus	557/298871.90%	31/5006.20%	24/5004.80%	23/500 4.60%	30/500 6.00%	none	**40/5008%**
UV-B r	GO:0009416 response to light stimulus	557/298871.90%	**34/5006.80%**	24/5004.80%	24/500 4.80%	22/5004.40%	none	none
Cold s	GO:0009409 response to cold	276/298870.90%	62/500 12.40%	59/500 11.80%	44/500 8.80%	34/500 6.80%	**54/50019.8%**	57/50011.4%
Cold r	GO:0009409 response to cold	276/29887 0.90%	**67/500 13.40%**	67/500 13.40%	43/500 8.60%	33/500 6.60%	56/50011.2%	48/5009.6%
Heat s	GO:0009408 response to heat	140/29887 0.50%	67/500 13.40%	59/50011.8%	45/500 9.00%	30/500 6.00%	**97/50019.4%**	59/50011.8%
Heat r	GO:0009408 response to heat	140/298870.50%	80/500 16.00%	91/500 18.20%	43/500 8.60%	28/500 5.60%	**93/50018.6%**	52/50010.4%
Osmotic s	GO:0006970 response to osmotic stress	474/29887 1.60%	91/50018.20%	94/500 18.80%	55/500 11.00%	29/500 5.80%	**95/50019%**	55/50011%
Osmotic r	GO:0006970 response to osmotic stress	474/298871.60%	**80/500 16.00%**	79/500 15.80%	39/500 7.80%	27/500 5.40%	68/50013.6%	47/5009.4%

‘s’ denotes shoot samples; ‘r’ denotes root samples.

#### 3.2 Results for tumor datasets

Two tumor datasets were also analyzed to verify the performance of the proposed method. The medulloblastoma dataset contains 34 samples, which can be divided into 25 tumor and 9 normal tissue samples, and assesses the expression of 5893 genes [41]. The leukemia dataset consists of 5000 genes and 38 samples [42]. The samples include 27 tumor and 11 normal tissue samples.

To make a fair comparison, all the six methods extracted 100 genes as characteristic genes from the two tumor datasets. The Gene Ontology (GO) enrichment and functional annotation of the extracted genes by all six methods was performed by *ToppFun*, which is publicly available at [http://toppgene.cchmc.org/enrichment.jsp].

Tables 6 and 7 list the top 10 closely related terms with P-value corresponding to different methods for the two tumor datasets. From Table 6, it can be seen that NMF-*L*_2,1_ outperforms the other three methods for all terms. For example, for the term M11197 in Table 6, the P-value from NMF-*L*_2,1_ is 7.36E-129 and 70/389 denotes that NMF-*L*_2,1_ extracts 70 genes corresponding to M11197, whereas NMFSC, PMD,CIPMF, PRFE and SPCA extracted 62, 62,48, 52 and 55 genes corresponding to M11197, respectively, and the total number of genes corresponding to M11197 was 389. In Table 7 we can see that our method outperforms than other method in seven terms, only in other three terms(17092989, 19755675, 11108479) PRFE have a lower P-value than our method. Thus, we can conclude that our method extracts more genes than others.

**Table 6:** P-value Terms for the Medulloblastoma Dataset.

ID	Name	P-value Sample frequency					
		NMF-L_21_	NMFSC	PMD	SPCA	CIPMD	PRFE
M11197	Housekeeping genes identified as expressed across 19 normal tissues	**7.63E-129 70/389**	3.28E-121 62/389	5.59E-92 59/389	6.28E-81 55/389	1.227E-6848/389	7.112E-7552/389
12456497	Human Leukemia Durig03 88 genes	**1.35E-11851/81**	1.29E-99 43/81	3.26E-77 38/81	4.90E-79 39/81	1.592E-7738/81	2.331E-5831/81
GO:0022626	Cytosolic ribosome	**9.06E-111 54/96**	8.62E-81 37/96	3.03E-56 32/96	7.30E-68 37/96	1.152E-3825/96	2.955E-4528/96
GO:0006415	Translational termination	**8.43E-108 53/95**	7.19E-78 37/95	6.91E-56 32/95	9.59E-68 37/95	1.388E-3526/95	2.146E-3426/95
GO:0006414	Translational elongation	**1.35E-105 56/130**	1.86E-77 40/130	2.04E-57 35/130	7.25E-66 39/130	1.311E-4935/130	6.78E-3628/130
GO:0006614	SRP-dependent cotranslational protein targeting to membrane	**7.66E-104 53/107**	5.26E-75 37/107	6.38E-54 32/107	2.18E-65 37/107	4.160E-5432/107	1.499E-4026/107
GO:0044391	ribosomal subunit	**1.86E-99 55/148**	1.54E-70 38/148	1.97E-51 33/148	7.75E-62 38/148	1.317E-3023/148	3.727E-3928/148
GO:0003735	Structural constituent of ribosome	**4.11E-95 54/156**	3.35E-67 37/156	2.38E-48 32/156	8.85E-59 37/156	9.511E-4633/156	3.656E-3427/156
GO:0005198	Structural molecule activity	**5.09E-65 58/641**	1.07E-54 44/641	9.98E-37 38/641	2.68E-44 43/641	1.952E-2628/641	1.192E-2935/641
GO:0003723	RNA binding	**9.48E-57 63/1568**	2.16E-39 47/1568	7.72E-26 41/1568	2.37E-30 45/1568	7.033E-2037/1568	1.153E-2037/1568

**Table 7:** P-value Terms for the Leukemia Dataset.

ID	Name	P-value sample frequency					
		NMF-L21	NMFSC	PMD	SPCA	CIPMD	PRFE
M11197	Housekeeping genes identified as expressed across 19 normal tissues.	**3.65E-41 40/389**	3.49E-40 33/389	1.57E-30 21/389	5.62E-23 23/389	2.584E-1820/389	1.05E-3230/389
17092989	Human Lymphoma Foge l07 33 genes	1.20E-33 18/33	6.25E-29 14/33	3.49E-32 12/33	2.57E-19 10/33	3.93E-2212/33	**8.43E-5123/33**
19755675	Human Leukemia Li09 419 genes	4.55E-25 25/410	4.27E2220/410	4.48E-21 18/410	none	6.82E-2223/410	**5.88E-2928/410**
19699293	Human Leukemia Bienkowska09 80 genes	**2.17E-22 14/75**	2.88E-15 9/75	8.91E-16 11/75	1.95E-13 9/75	2.58E-1712/75	3.03E-1712/732
15474998	Mouse StemCell Lindmark04 950 genes	**4.71E-21 24/732**	2.45E-20 22/732	4.89E-18 20/732	1.52E-15 20/732	none	none
16872506	Human Leukemia Yukinawa06 2000 genes	**1.54E-20 33/1505**	4.05E-18 28/1505	1.10E-16 23/1505	none	none	none
18689800	Human EmbryonicStemCell Thomas08 1088 genes	**1.97E-20 29/1023**	1.06E-17 26/1023	4.59E-19 21/1023	1.03E-10 20/1023	2.28e-1121/1023	3.62E-1222/1023
11108479	Human Leukemia Ben-Dor00 143 genes	1.22E-18 14/129	1.17E-15 11/129	1.10E-16 12/129	1.61E-15 12/129	1.424E-1714/129	**1.45E-2217/129**
12077300	Human Lymphoma Lossos02 152 genes	**5.43E-17 13/99**	6.91E-14 10/99	8.21E-16 10/99	3.41E-129/99	none	none
M11620	Genes induced in the liver during hepatitis B (HBV) viral clearance in chimpanzees.	**3.20E-15 12/101**	3.27E-12 9/101	3.58E-129/101	4.18E-12 8/101	6.96E-119/101	5.68E-1411/101

In summary, we conclude that our method is generally superior to others and is effective for the extraction of genes.

## 4 Conclusions

In this paper, we proposed an effective method to select characteristic genes with*L*_2,1_-norm minimization of both the error function and the regularization term. The *L*_2,1_-norm-based error function is robust to outliers and noise in the data points and is computationally efficient. Furthermore, the *L*_2,1_-norm-based regularization term is used to generate a sparse solution. We also used the nonnegative factorization method to avoid the problems stemming from the high-dimensional and nonnegative nature of the data. In summary, our method can cope with high dimensionality, non-negativity, sparseness and noise simultaneously.

Furthermore, the genes selected by our method and others from both plant and tumor datasets were compared using GO enrichment. These results indicate that the proposed NMF-*L*_2,1_ method is superior to SPCA and PMD for selecting characteristic genes.

## Supporting Information

S1 Table The plant gene expression dataset. (ZIP)
